# Efficiency-fairness trade-offs in evacuation management of urban floods: The effects of the shelter capacity and zone prioritization

**DOI:** 10.1371/journal.pone.0253395

**Published:** 2021-06-22

**Authors:** Woi Sok Oh, David J. Yu, Rachata Muneepeerakul

**Affiliations:** 1 Department of Agricultural and Biological Engineering, University of Florida, Gainesville, FL, United States of America; 2 Lyles School of Civil Engineering, Purdue University, West Lafayette, IN, United States of America; 3 Department of Political Science, Purdue University, West Lafayette, IN, United States of America; 4 Center for the Environment, Purdue University, West Lafayette, IN, United States of America; Al Mansour University College-Baghdad-Iraq, IRAQ

## Abstract

With increasing flood risk, evacuation has become an important research topic in urban flood management. Urban flood evacuation is a complex problem due to i) the complex interactions among several components within a city and ii) the need to consider multiple, often competing, dimensions/objectives in evacuation analysis. In this study, we focused on the interplay between two such objectives: efficiency and fairness. We captured the evacuation process in a conceptual agent-based model (ABM), which was analyzed under different hard infrastructure and institutional arrangement conditions, namely, various shelter capacity distributions as a hard infrastructure property and simultaneous/staged evacuation as an institutional arrangement. Efficiency was measured as the time it takes for a person to evacuate to safety. Fairness was defined by how equally residents suffered from floods, and the level of suffering depended on the perceived risk and evacuation time. Our findings suggested that efficiency is more sensitive to the shelter capacity distribution, while fairness changes more notably according to the evacuation priority assigned to the divided zones in staged evacuation. Simultaneous evacuation generally tended to be more efficient but unfairer than staged evacuation. The efficiency-fairness trade-off was captured by Pareto-optimal strategies, among which uniform capacity cases led to a higher efficiency while prioritizing high-risk residents increases fairness. Strategies balancing efficiency and fairness featured a uniform capacity and prioritized high-risk residents at an intermediate time delay. These findings more clearly exposed the interactions between different factors and could be adopted as benchmarks to inform more complicated evacuation ABMs.

## Introduction

Currently, increased hydrological extremes have reduced the resilience of urban areas to floods [1,2]; residents must escape urban areas within a short period [3–5]. Recent years have witnessed the evacuation of urban areas worldwide as the result of the threat of floods, e.g., Beichuan County, China, in 2008, Oroville, California, in 2017, and Manno, Japan, in 2018. Urban flood evacuation has a direct implication on the survival and welfare of humans during or before floods. For instance, Hurricane Rita arrived in Texas on September 24, 2005, forcing its residents to evacuate. The hurricane resulted in terrible traffic congestion and insufficient fuel supply issues during the evacuation of Texas residents, one of the most populous states in the US [6]. Many researchers have explored evacuation problems to determine ways to improve urban flood resilience.

Despite the complexity of the evacuation process, evacuation has often been studied only in terms of efficiency, focusing on reducing evacuation times. This perspective is incomplete. Following an increasing number of researchers integrating social dimensions into disaster management [7,8], this study incorporates fairness into evacuation process evaluation. It would be straightforward if a policy improves both efficiency and fairness: a policymaker could easily select such a win-win strategy. However, trade-offs may exist between efficiency and fairness [9], presenting a policy dilemma for policymakers. Although evacuation research has examined fairness from diverse perspectives [10,11], the interplay between efficiency and fairness has not been fully addressed.

Efficiency and fairness in the flood-induced evacuation of urban areas are influenced by the social, hydrological, hydraulic, technical, and traffic characteristics of a city [11,12]. In particular, the evacuation problem involves hard infrastructure that physically protects or accommodates people against natural hazards (e.g., shelters, roads) [13]. Evacuation studies have optimized the properties of shelters (e.g., location, capacity, and number) to achieve the best performance, with a typical focus on reducing evacuation times [3,9,14–18]. The shelter capacity particularly refers to the maximum number of residents that a shelter can accommodate. Previous studies have demonstrated that the shelter capacity is critical in successful flood evacuation management [3,19–21]. These studies often defined the shelter capacity as a constraint in location-allocation models of the evacuation problem [16,22,23] (although the shelter capacity may be less critical under certain circumstances, e.g., when many residents remain at home or evacuate to high places instead of to shelters [24–26]). Under multi-shelter conditions, the capacity could either remain the same or vary among shelters. The shelter capacity distribution may affect evacuation outcomes differently.

The problem of evacuation is also related to institutional arrangements that intangibly facilitate successful evacuation (e.g., contraflow or staged evacuation). Institutional arrangements are socially constructed rules, norms, and strategies that regulate human decisions and interactions [27,28]. These institutional components are also important in strategic evacuation design because they often operate in conjunction with hard infrastructure and influence the system response to floods [29]. Recent studies have addressed different institutional arrangements in evacuation models [30–35]. Some studies have explored simultaneous and staged evacuation planning as one institutional arrangement during evacuation. Chen and Zhan [32], for example, applied an agent-based model (ABM) to investigate the effects of simultaneous and staged evacuation planning under different road structures and zone priorities. This study was limited to examining the effects only in terms of efficiency. Xie et al. [35] modified a staged evacuation algorithm under a multi-exit setting, focusing on total evacuation time minimization. Liu et al. [31] designed a cell-based network model to gain deep insights into staged evacuation features. Many evacuation studies, however, have focused solely on either hard infrastructure or institutional arrangements though there are some models, such as the Life Safety Model (LSM), which incorporate both sides (please refer to Refs [24–26,36–39]). Addressing the interplay between efficiency and fairness in urban flood evacuation requires an approach that integrates both the hard infrastructural and institutional aspects of the problem.

This paper aims to assess how hard infrastructure (the shelter capacity distribution) and institutional arrangements (simultaneous vs. staged evacuation) interact to yield trade-offs between efficiency and fairness in urban flood evacuation. Our contribution is a clear picture of the interplay between efficiency and fairness, not a development of realistic, complex models. To this end, we developed an evacuation ABM with a simple design such that the interaction between hard infrastructure and institutional arrangements is easy to interpret (please refer to the ODD Protocol in S1 File for more detail). With the use of this ABM, we ask the following questions: How do the shelter capacity distribution and simultaneous/staged evacuation affect the efficiency and fairness of urban flood evacuation? What is the interplay between efficiency and fairness? Is there a single best strategy when considering multiple dimensions in evacuation?

## Model design

Many evacuation studies have adopted ABMs [17,18,40] because of their distinct strengths over other modeling methods. Firstly, ABMs enable us to focus on individuals and their heterogeneous evacuation behaviors in a bottom-up approach [41]. As such, we may better understand how the decisions and behaviors of evacuees result in systematic patterns. Second, ABMs simplify local rules of certain behavioral patterns to solve transportation problems with various elements [33,42]. ABMs fit well into evacuation modeling, as traffic rules also highly impact transportation simulations [43]. Finally, institutional and physical infrastructural components are readily encompassed in ABMs [44,45].

**Fig 1:**
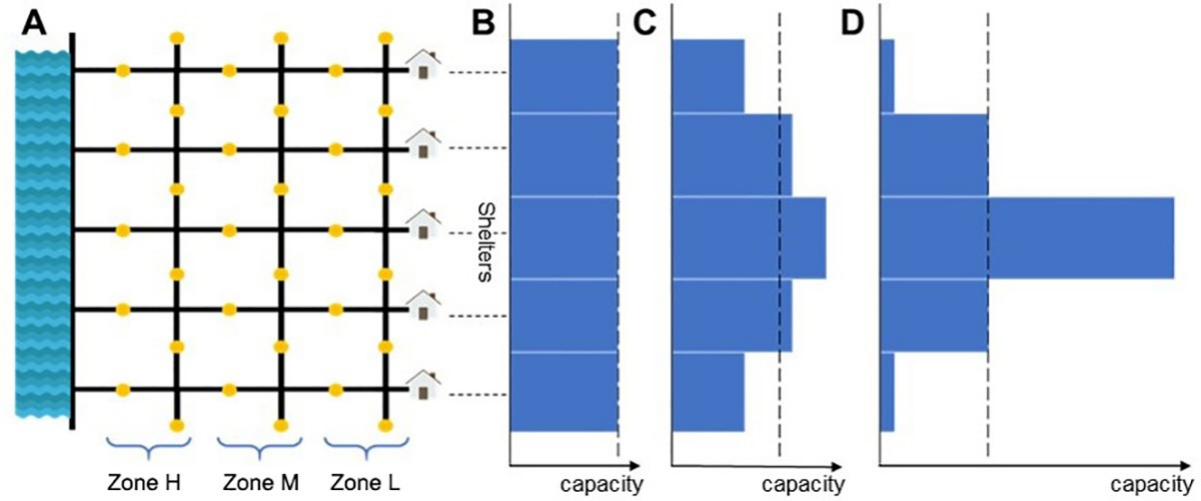
Overview of the model setup. (A) Evacuees are generated at the sources (yellow circles) and head to the nearest shelters at the right (the house shapes) via roads. The left side comprises flood-prone areas. The closer an area to the water, the higher the risk is. Zone H is a high-risk area, and Zone L is a low-risk area. (B-D) Different shelter capacities are set as hard infrastructure properties. The shelter capacity of B is uniform, that of C is moderately nonuniform, and that of D is strongly nonuniform. The dashed lines in B-D indicate the guideline of a uniform capacity.

For clarity of analysis, many evacuation-related factors were omitted (e.g., signal control, police intervention, etc.) in our ABM (Fig 1). The form of the road network was a simple grid. The modeled city was divided into three risk zones based on their distance to the waterfront. Five shelters were placed at the opposite end of the city, away from the waterfront, and the capacities of the shelters followed one of three sets of values, representing different levels of capacity uniformity. During evacuation, the car speed remained constant. Simultaneous and staged evacuation plans with a range of time delays between zones were compared. Details of the different model components are described in the following subsections. An ODD Protocol of our ABM is provided in S1 File.

### Model setup

The NetLogo software was used to develop the ABM. In NetLogo, the modeled world is two-dimensional and is divided up into a grid of patches. Each patch is a square piece of “ground,” and cars can move over each patch. A tick counter is used in NetLogo to represent the passage of simulated time. Our ABM was designed to describe a city whose roads occur in a grid-network form (Fig 1A) [32,46–49]. This type of gridded conceptual environment has been applied in previous evacuation research [32,41,50]. The left boundary of the model comprised flood-prone areas (the blue areas in Fig 1A). Residents perceived a greater flood risk with decreasing distance to these areas [51,52]. The right boundary was safer from floods, and there were five shelters for evacuation. The risk levels of residents were assumed to be proportional to the distance between their residence and the above shelters (the right border in Fig 1A). Residents started their evacuation processes in their vehicles from sources located in the middle of the roads (the yellow points in Fig 1A)—adapted from the source-sink concept in other network-based evacuation studies [53]. Agents were equally distributed across all the source patches (a total of 1500 agents) and evacuated to one of the shelters (sinks; the dark blue patches in Fig 1A). They were assumed not to remain at home nor move to higher ground but evacuate to shelters in their cars.

### Shelters (hard infrastructure)

Shelters can accommodate a certain number of residents at maximum (the shelter capacity). By varying the capacity distribution, we forced evacuees to make decisions based on different configurations of the distance between their residence and the closest shelter. We set five shelters on the opposite side from the waterfront (Fig 1A). Ninety percent of residents were assigned to the nearest shelter (full information). The remaining residents did not possess information regarding the closest shelter and randomly evacuated to one of the shelters. After arriving at a fully occupied shelter (no information was provided during the car ride to a shelter), residents would search for the closest shelter with accommodation space assuming that shelters shared capacity status information. Given the same total population, there were three possible forms of shelter capacity: uniform (U: 303 people per shelter, as shown in Fig 1B), moderately nonuniform (MN: 204, 337, 433, 337, and 204 people across the five shelters; similar to a normal distribution with a high variance, as shown in Fig 1C), and strongly nonuniform capacities (SN: 41, 304, 825, 304, and 41 people across the five shelters; similar to a normal distribution with a low variance, as shown in Fig 1D). These capacity distributions, while simplistic, allowed us to explore the interactions between the multiple objectives. The shelter locations, numbers, and capacities remained the same throughout the evacuation process. The sum of the five shelter capacities was equal to the total population with an additional 1% to prevent possible numerical errors (1515 in total). When a shelter was fully occupied, the connecting road to this shelter was closed, thus preventing evacuee inflow.

### Institutional arrangement

**Fig 2:**
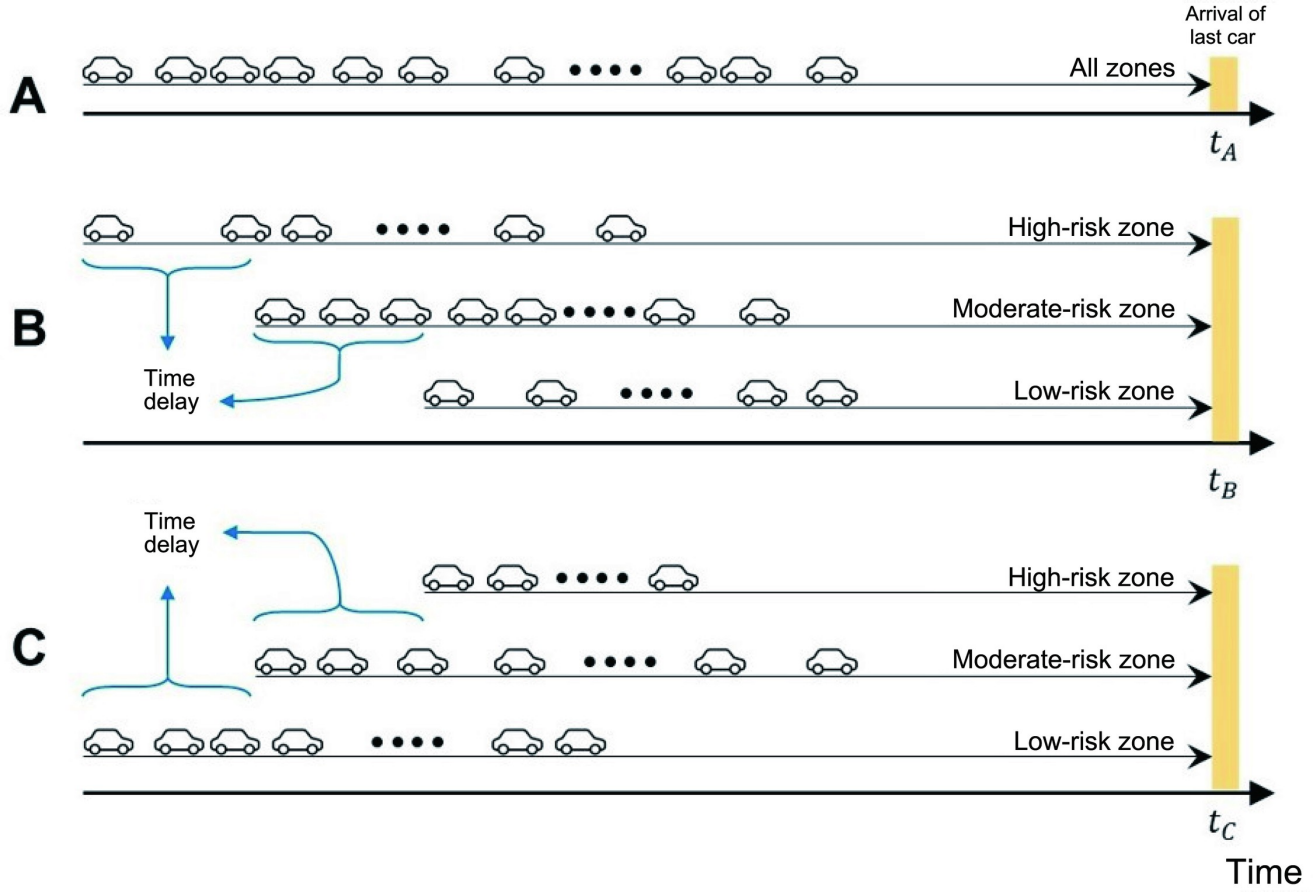
Schematic diagram of the various evacuation institutional arrangements. (A) Simultaneous evacuation, (B) staged evacuation in order of high-risk, moderate-risk, and low-risk zones (HML), and (C) staged evacuation in order of low-risk, moderate-risk, and high-risk regions (LMH). The black cars are the individual agents departing at the corresponding times. A yellow line indicates the final arrival time of the last evacuee. *t*_*A*_, *t*_*B*_, and *t*_*C*_ denote the evacuation durations in A, B, and C, respectively. The blue ranges denote the time delays between the various zones.

To investigate the effects of the institutional arrangement, we compared simultaneous evacuation and staged evacuation plans (or strategies). Regarding the staged evacuation plan, we considered a range of time delays between the evacuation processes of the different zones. In simultaneous evacuation, evacuees simultaneously started their evacuation process from their origins. The term “simultaneous” refers to the fact that the first evacuating agent in each zone was allowed to start at the same time, not that all agents started their evacuation process at the same time. In the cases of the staged evacuation strategies, we divided the city into high-risk (H), moderate-risk (M), and low-risk zones (L). For ease of reference, we referred to each staged evacuation plan by its zone priority: for example, under the HML strategy, high-risk residents evacuated first, moderate-risk residents began evacuating after a certain time delay, and low-risk residents finally began evacuating after another time delay. Six different zone sequences—HML, LMH, HLM, MHL, MLH, and LHM—covering a range of time delays (the blue ranges in Fig 2) were investigated to determine their effects on the efficiency and fairness of the evacuation process [32].

### Agent behavior

The path decisions of individuals play a large role in evacuation planning [16]. Agents, represented by the evacuating residents in their vehicles, traveled to the closest shelter from their origins (sources). When an agent perceived multiple equidistant shelters, he/she randomly chose between them. To simplify the model, we assumed that an evacuee possessed full information to arrive at a destination under the shortest path. Evacuees randomly chose one of the shortest paths to head toward their destinations.

**Fig 3:**
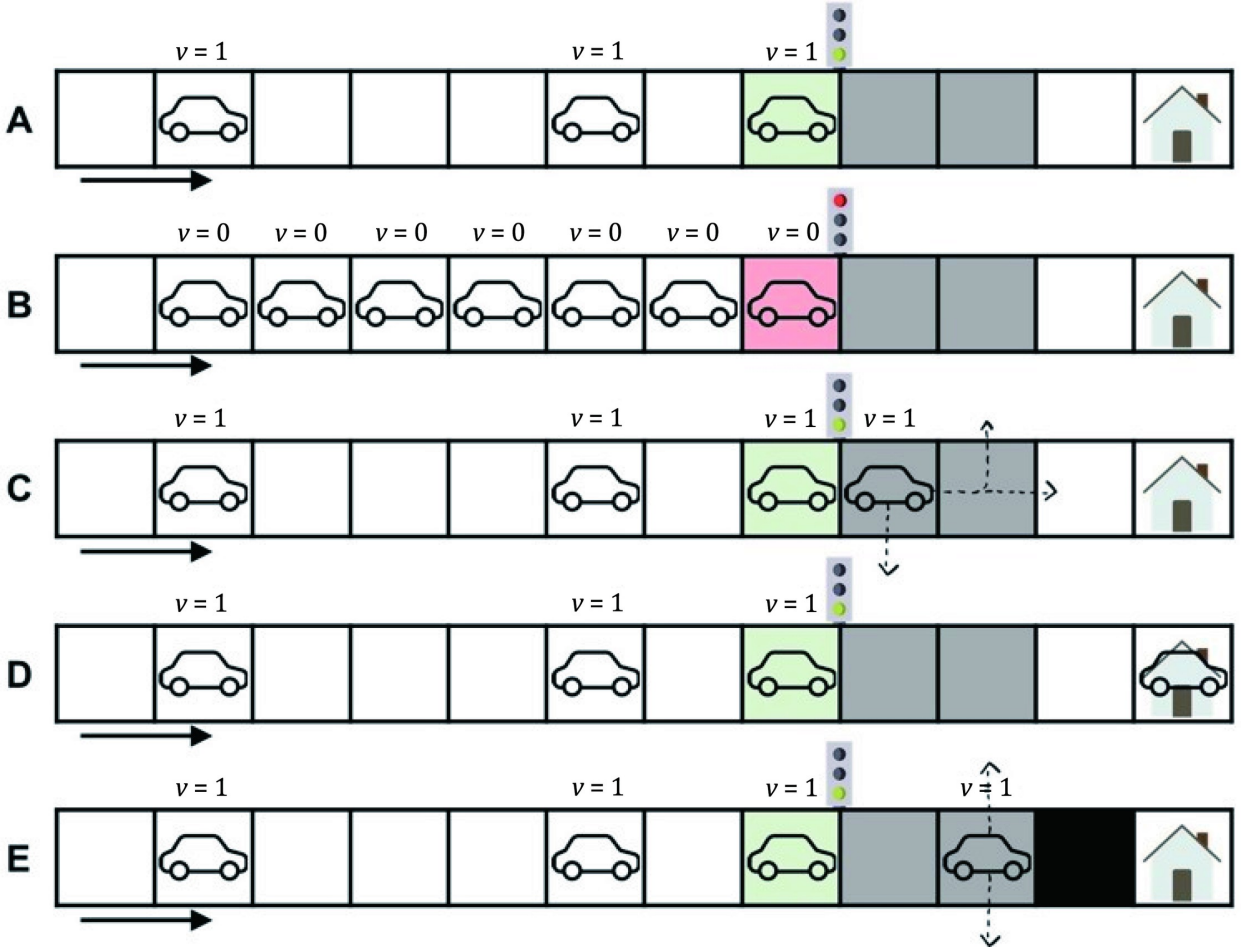
Five cases of simplified car movement at two speeds: Zero and one. The green, yellow, or red areas represent traffic lights. The white areas are roads, and the gray areas are intersections. When a car arrives at a shelter, the evacuation process is completed.

Evacuees entered the road and started their trips based on previously selected routes. Traffic movement in this model followed a simplified version of a cellular automaton traffic model (based on the Nagel-Schreckenberg traffic model [54]) at a fixed speed. This setting eased traffic congestion since ABM studies pointed out that a speed difference between adjacent cars causes traffic congestion [55,56]. After agents started moving, they encountered five different situations. Firstly, agents moved at speed 1 when they were not blocked by stationary cars right ahead, and the traffic light was green (Fig 3A). If there was a stationary car ahead or the traffic light was red, a car stopped (Fig 3B). Agents either continued straight, turned right, or turned left at intersections based on their randomly selected routes (Fig 3C). When a shelter was not full, the agent entered the shelter, and the evacuation process was completed (Fig 3D). If the shelter was full, the road was closed. Then, the agent searched for a new shelter with accommodation space and headed to this new shelter (Fig 3E).

### Quantifying efficiency and fairness

We performed 500 simulations for each combination of hard infrastructural and institutional properties and statistically analyzed the results in the efficiency and fairness dimensions. The evacuation duration (*d*) is an indicator of the efficiency of a given strategy (unit: tick). We calculated the evacuation time for the final evacuee arriving at the shelter in each simulation and determined the average value:

$$d=\frac{1}{500}\sum_{k=1}^{500}\max(t_{k}),$$ (1)

where **t**_**k**_ is a vector of the evacuation times for all agents in the *k*^th^ simulation. A shorter duration indicated a more efficient evacuation strategy. To study fairness, we introduced the degree of unfairness (DOU) to estimate how unequally each resident suffered from floods. First, we defined the degree of suffering of an agent *i* (*s*_*i*_) to quantify how much the agent suffered from floods. This term depended on both the risk level of the residence of agent *i* and the time required to reach a safe shelter. *s*_*i*_ was derived as follows:

$$s_{i}=t_{i}\cdot r_{i},$$ (2)

where *t*_*i*_ is the evacuation duration, i.e., the time required to reach a shelter, for agent *i*, and *r*_*i*_ is the initial flood risk of agent *i*, proportional to the distance between the residence of agent *i* (the initial location) and the safest areas where the shelters are located (the right side in Fig 1A). For example, an agent living near a shelter felt safe and attained a lower risk value. *r*_*i*_ was sometimes correlated to *t*_*i*_ if there was no institutional arrangement. Otherwise, *r*_*i*_ and *t*_*i*_ could behave differently depending on the configured institutional setting. The Gini coefficient has often been considered to quantify wealth disparity in economics and has been applied to diverse fields [57,58]. To calculate the DOU, we determined the Gini coefficients of every *s*_*i*_ and calculated the average value over 500 simulations:

$$DOU=\frac{1}{500}\sum_{k=1}^{500}Gini(s_{k})=\frac{1}{500}{\sum_{k=1}^{500}\left(\frac{\sum_{i=1}^{n}\sum_{j=1}^{n}\left|s_{i}-s_{j}\right|}{2n\sum_{i=1}^{n}s_{i}}\right)}_{k},$$ (3)

where *n* is the number of initial residents (*n* is set to 1500), and **s**_**k**_ is a vector of *s*_*i*_ in the *k*^th^ simulation (we use **s** to represent the **s**_**k**_ values in the general cases). The lower the DOU is, the fairer the evacuation process. Although the DOU contains a *t*_*i*_ term, it is uncorrelated with the efficiency due to the Gini coefficient calculation. The DOU values vary between 0 and 1 (dimensionless).

### Pareto-optimal strategies

The Pareto optimality was first established in economics to study economic efficiency and income distribution [59]. This concept has been applied to diverse problems in hydrology, mechanical engineering, and electrical engineering [60–63]. The Pareto optimality has often been used to capture trade-offs between multiple objectives. A strategy is said to be a Pareto-optimal strategy if no alternative exists that improves one dimension without worsening another. Specifically, within the context of our model, an evacuation strategy is said to exhibit Pareto optimality if no other strategies achieve a shorter evacuation time without being unfairer or achieve a greater fairness without requiring a longer evacuation duration. The set of Pareto-optimal strategies is sometimes referred to as the Pareto frontier.

## Results and discussion

### Effects of the shelter capacity distribution and institutional arrangement on efficiency and fairness

**Fig 4:**
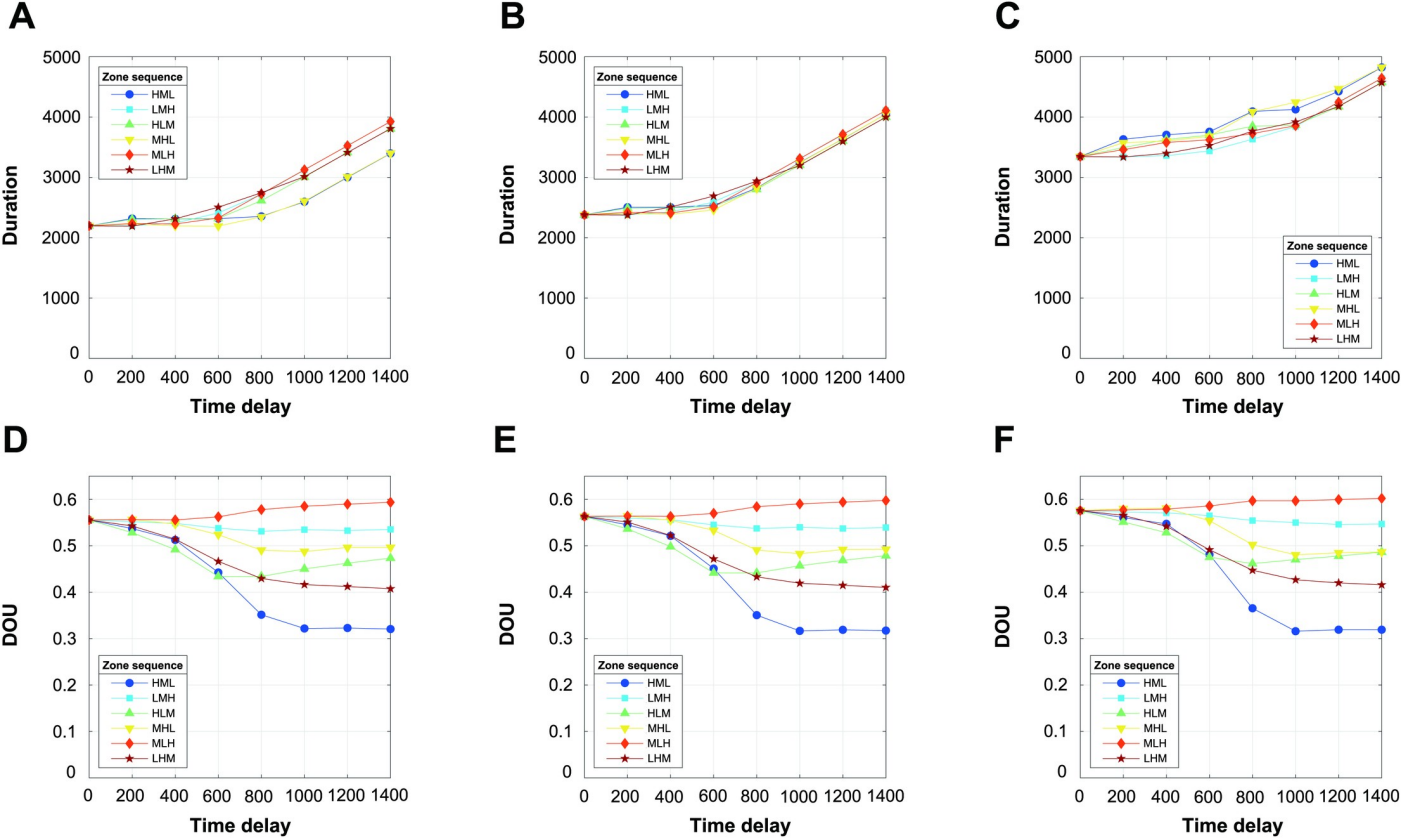
Mean evacuation duration and mean DOU, averaged over 500 simulations, as a function of the time delay under the different shelter capacity distributions and institutional arrangements. A time delay of zero indicates simultaneous evacuation. Each column represents a different shelter capacity: (A, D) uniform, (B, E) moderately nonuniform, and (C, F) strongly nonuniform.

Our model results suggested that the evacuation duration (efficiency) is more sensitive to the shelter capacity distribution than is the DOU (fairness). The evacuation process lasted longer under nonuniform shelter capacity distribution conditions (compare Fig 4A–4C). However, the DOU was minimally impacted, revealing only slight increases when the time delay was short (compare Fig 4D–4F). Under nonuniform capacity conditions, shelters with a lower capacity became fully occupied soon, forcing those who arrived thereafter to travel further to find a shelter with accommodation space. This led to a longer evacuation time and greater differences between the early and late arrivals, with the former being more pronounced than the latter. The dependence of the evacuation time on the shelter capacity distribution was also mediated by the institutional arrangement. The low-risk-low-priority strategies (HML and MHL) were the most sensitive, and they were the most efficient under the uniform capacity distribution (Fig 4A) and the least efficient under the strongly nonuniform capacity distribution (Fig 4C).

Note that the slopes between the time delay and duration converged to 2 at large time delays (> 1000) (the slope was calculated as (duration(*t*_2_)−duration(*t*_1_))/(*t*_2_−*t*_1_)). This behavior stemmed from our model structure comprising three zones (i.e., zones H, M, and L in Fig 1A). At large time delays, the evacuation duration was dominated by the total time delay, the sum of the two delays between the first and second zones and between the second and third zones. Note also that the total time delay constituted the lower bound of the evacuation duration. In addition, the strategies with the same last zone in their sequences (HML/MHL, LMH/MLH, HLM/LHM) resulted in the same evacuation duration at large time delays. At large time delays, the residents in these three zones did not encounter each other, and the evacuation duration depended on the residents of the last zone. These very long time delays, while theoretically interesting as limiting scenarios in the model, may be less relevant in practice.

The DOU was more sensitive to the institutional arrangement than was the evacuation duration: the different zone sequences resulted in highly different relationships between the DOU and time delay (Fig 4D–4F). The staged evacuation plans generally resulted in lower DOUs and longer durations (less efficient but fairer) over the simultaneous evacuation plans (zero time delay), with the exception of the MLH strategy. Under the MLH strategy, the DOU increased with increasing time delay because the high-risk residents suffered disproportionately more than did the other residents. As one may expect, the two most unfair strategies prioritized the high-risk residents last (MLH and LMH), while the HML plan was among the fairest strategies.

Under the HLM strategy, we observed an unexpected relationship between the DOU and time delay. The DOU first decreased with increasing time delay, similar to the other cases (< 600), reached a plateau at intermediate time delays (600–800), and then increased with a further increase in the time delay (> 800). At short time delays, the HLM strategy prioritized the high-risk residents, which resulted in them suffering less. Thus, the evacuation process became fairer with increasing time delay. At long time delays, the high-risk residents continued to suffer less. However, the moderate-risk residents, prioritized the least during evacuation, suffered greatly from the increasing time delays (longer evacuation duration), leading to unfairer evacuation conditions.

**Fig 5:**
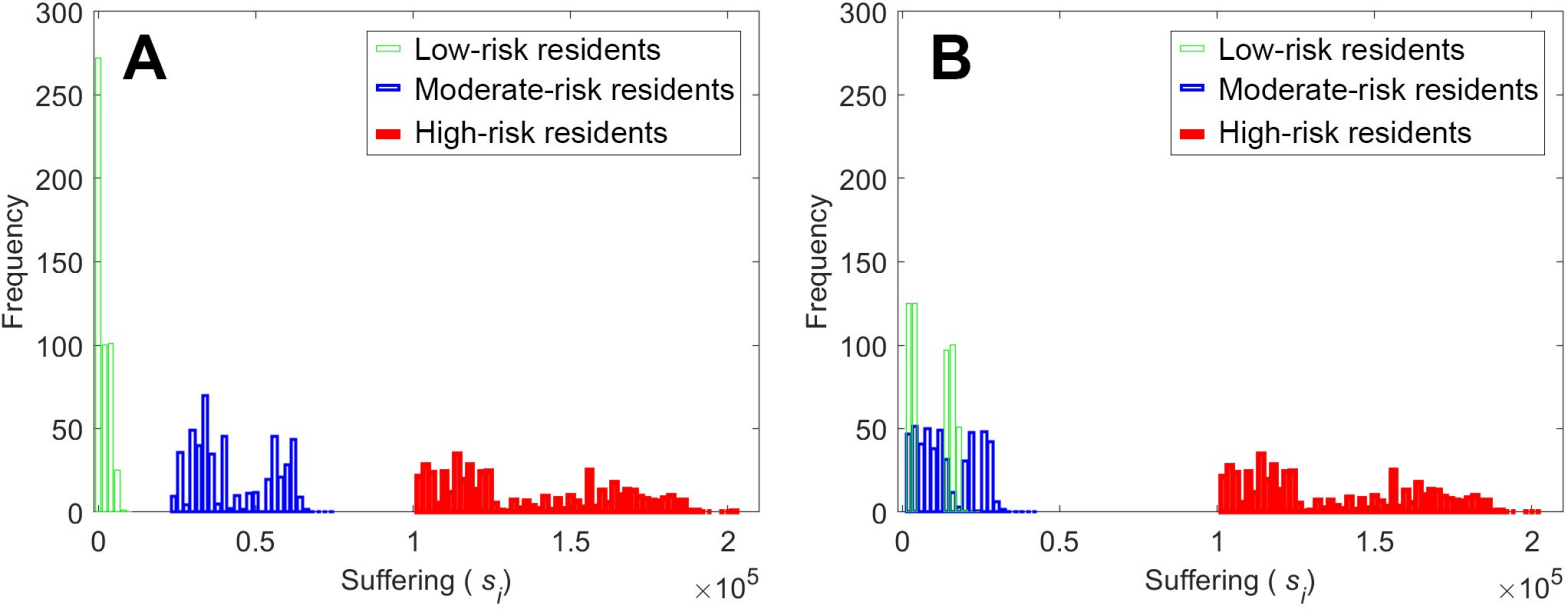
Distributions of the suffering levels for 1500 residents under the different zone sequences: (A) LMH strategy and (B) MLH strategy. Two cases are compared under a time delay of 1000 and a moderately nonuniform shelter capacity. The color represents the different zones divided in staged evacuation.

Another unexpected result was that while the LMH strategy should theoretically attain the highest DOU (unfairest), it was the MLH strategy that yielded the highest DOU. Under the LMH strategy, Zone L residents suffered the least, while Zone H residents suffered the most, with Zone M residents experiencing a moderate suffering degree. In particular, the suffering degree of the population can be divided into three distinct groups (Fig 5A). By allowing the Zone M residents to evacuate first, the MLH strategy reduced their suffering without substantially increasing the suffering degree of the Zone L residents. This likely occurred because at the first stage of evacuation, there were still numerous shelters with accommodation space, so even under the delayed start, the Zone L residents quickly found a shelter. As a result, the trimodal distribution of the suffering degree under the LMH strategy became a more polarized bimodal distribution (Fig 5B), hence leading to a higher DOU.

### Pareto-optimal strategies

**Fig 6:**
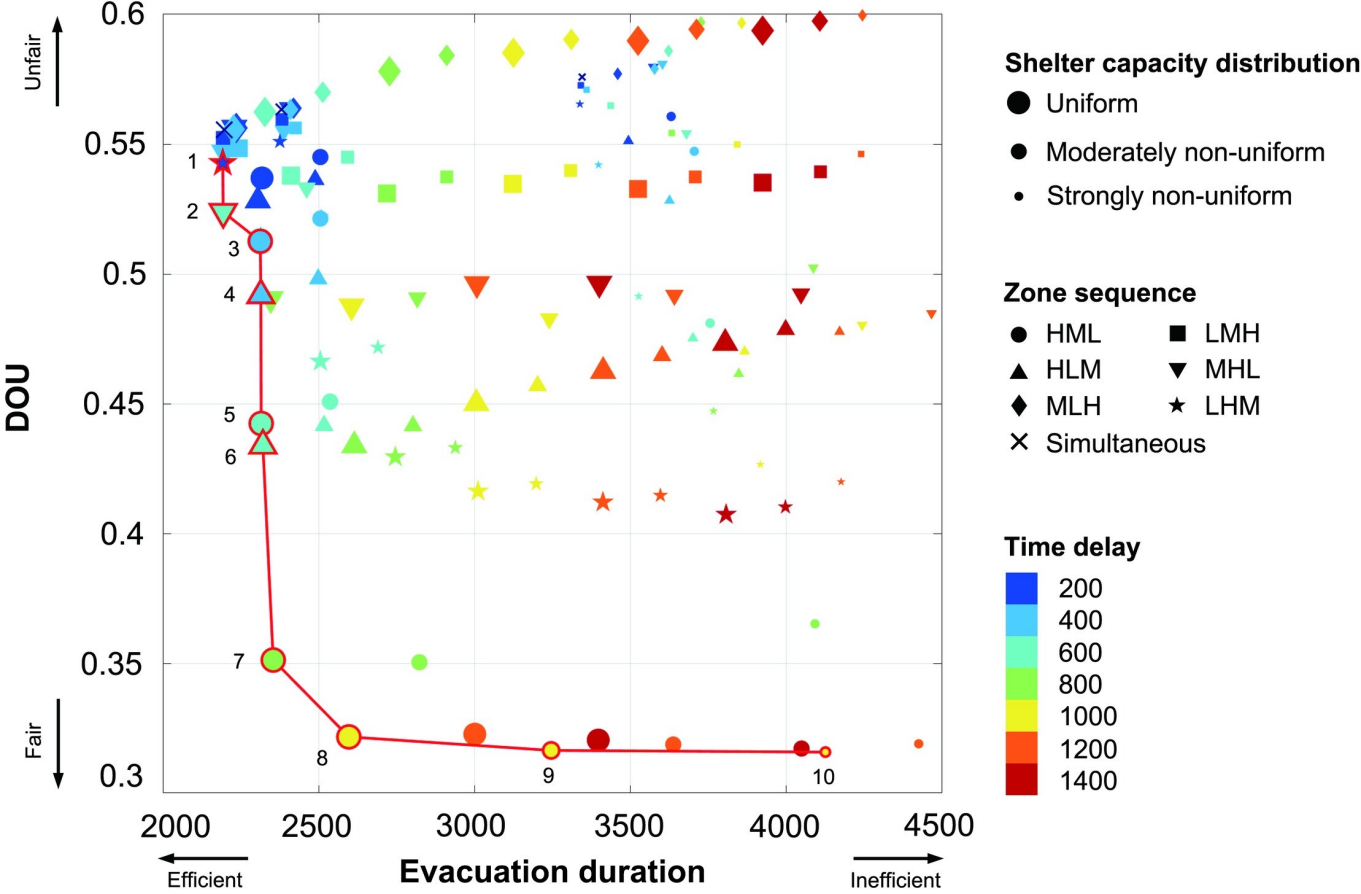
Pareto-optimal strategies considering efficiency (evacuation duration) and fairness (DOU). Each dot represents the mean outcomes of the evacuation duration and DOU under the different settings of hard infrastructure and institutional arrangement properties (500 simulations per dot; a total of 129 dots). The marker sizes indicate the different hard infrastructure properties (the shelter capacity distributions). The shapes indicate the different zone sequences, and the colors indicate the varying time delays. The highlighted dots with red edges are the Pareto-optimal strategies, forming a Pareto frontier (connected by red lines). The 10 Pareto-optimal strategies are as follows: 1 = LHM-200-U; 2 = MHL-600-U; 3 = HML-400-U; 4 = HLM-400-U; 5 = HML-600-U; 6 = HLM-600-U; 7 = HML-800-U; 8 = HML-1000-U; 9 = HML-1000-MN; 10 = HML-1000-SN.

To facilitate discussion in this section, each staged evacuation plan was labeled after its zone priority in staged evacuation, the time delay between the various zones, and the shelter capacity distribution: for example, under the HML-200-U strategy, the high-risk residents evacuated first, the moderate-risk residents began evacuating after a delay of 200 time units, and the low-risk residents then began evacuating after another delay of 200 time units, with the five shelters exhibiting a capacity of 303 people. Our model results suggested that it is impossible to achieve a perfect strategy that maximizes both efficiency and fairness: only Pareto-optimal strategies are possible (Fig 6). Among the strategies explored, the most efficient was the LHM-200-U strategy (Dot 1). However, this strategy was among the unfairest plans, with a DOU value of 0.54. At the other extreme, the fairest strategy, the HML-1000-U plan (Dot 10), was among the most inefficient ones, with an evacuation duration > 4000. The Pareto frontier connected these two extremes (connecting Dots 1 through 10, Fig 6) with the Pareto-optimal strategies divided in two general groups: efficient-but-unfair (Dots 1–7, duration < 2500, DOU > 0.35) and fair-but-inefficient (Dots 8–10, DOU < 0.35, duration > 2500). The strategies that seemed to better balance efficiency and fairness were the HML-800-U and HML-1000-U strategies (Dots 7 and 8, respectively).

The eight most efficient Pareto-optimal strategies featured uniform shelter capacities (Dots 1–8). In contrast, the four fairest Pareto-optimal strategies featured the HML sequence (Dots 7–10). Particularly, those with nonuniform shelter capacities were the fairest but very inefficient (Dots 9–10). Regarding the zone sequence, eight out of ten strategies (Dots 3–10) prioritized the high-risk residents (two HLM and six HML strategies). Two exceptional cases emphasized efficiency (Dots 1 and 2). A greater time delay usually led to a lower efficiency among these Pareto-optimal strategies: the efficient-but-unfair group (Dots 1–7) featured time delays ranging from 200 to 800, while the fair-but-inefficient group (Dots 8–10) featured a time delay of 1000. Within the range of simplified ABM settings, these results suggested that combinations of a uniform shelter capacity and high-risk population priority yield balanced outcomes in terms of efficiency and fairness.

### Limitations

Fairness can be interpreted in several ways and is thus challenging to quantify. Here, we defined fair as denoting equal degrees of suffering among individuals, but there are other ways. Recent evacuation models have considered fairness using system optimal (SO) approaches, but their definitions of fairness are different [9–11]. However, other studies have related fairness to the priority given to those with the least resources. Therefore, one must understand the fairness definition within a given context. The inappropriate application of a certain metric to assess fairness in a different context could produce meaningless outcomes and ruin the usefulness of a model.

Our ABM is simple in design, as we aimed to isolate the effects of different factors and investigate their interplay more clearly. Admittedly, the ABM omitted many realistic components in real-world evacuation management, which thus limited its practical use. These limitations should be overcome with more complex ABMs that incorporate real-world data (e.g., population distribution, GIS maps, demographic variables, etc.) and various metrics (e.g., death rate, economic loss, etc.) to increase the model realism while applying our findings as benchmarks to evaluate the effects of these additional features. For example, an actual road map could be used in place of the simple grid to examine how it alters the nature of the efficiency-fairness trade-off. There could be disruptions to infrastructure (e.g., roads, power, controls, communications, etc.), and the evacuee behaviors could cause disorderly patterns during evacuation. Information could be limited in choosing where to evacuate, which could influence the emergent patterns of urban flood evacuation. Residents have different ways of evacuation: some evacuate by cars, while others walk to shelters or even become trapped [64]. Other relevant considerations include the evacuation rate, risk perception, death rate, and probability of remaining home. Regarding fairness, the spatial distribution and correlation of risk, wealth, and natural resources (a water body could provide resources but could also increase the flood risk) are important aspects that should be explored [65,66]. We should also assess how different strategies affect the interactions between resource mobilization and loss of life/property.

## Conclusion

In this study, we aimed to address the following research questions: How do the shelter capacity distribution and simultaneous/staged evacuation affect the efficiency and fairness of urban flood evacuation? What is the interplay between efficiency and fairness? Is there a single best strategy when considering multiple dimensions during evacuation? Our findings suggest that the shelter capacity distribution highly affects efficiency and slightly affects fairness. The institutional arrangement—simultaneous or staged evacuation—strongly controls fairness and mediates the sensitivity of efficiency to the shelter capacity distribution. Trade-offs complicate the decision-making process of policymakers in choosing evacuation strategies: a strategy could be efficient but unfair. A single best strategy that maximizes both efficiency and fairness does not exist. However, Pareto-optimal strategies are the best options.

More complex model features, e.g., less orderly road patterns or a spatial correlation between risk and wealth, may alter these results. Nevertheless, our results could serve as a benchmark against more complex ABMs by offering an opportunity to identify how any additional features contribute to differences in the outcomes. In the future, we will consider more realistic features based on the limitations described in the previous section and expand our work to contribute to a deeper understanding of urban flood evacuation.

## Supporting information

S1 File ODD protocol. (DOCX)
