# Supplementary material for: Efficiency-fairness trade-offs in evacuation management of urban floods: The effects of the shelter capacity and zone prioritization
Source: PLoS One. 2021 Jun 22;16(6):e0253395. doi: 10.1371/journal.pone.0253395 (PMC8219134; doi:10.1371/journal.pone.0253395)
Supplement: S1 File — (DOCX) [file pone.0253395.s001.docx]

**Supporting Information**

**S1 File. ODD protocol**

1. **Overview**

This material is an ODD (Overview, Design concepts, and Details) protocol to supplement our ABM’s missing explanation from the manuscript.

1. **Purpose**

This conceptual ABM is built to study urban flood evacuation incorporating hard infrastructure and institutional arrangements. We aim to capture i) how shelter capacity distributions (related to hard infrastructure) and simultaneous/staged evacuation (related to institutional arrangements) influence the efficiency and fairness of urban flood evacuation, ii) what kind of interactions we observe between efficiency and fairness (e.g., win-win, trade-off, lose-lose), and iii) how Pareto-optimal strategies are formed in the efficiency-fairness plane.

1. **Entities, State variables, and scales**

The model environment represents a conceptual urban space with a waterfront area on the left side (blue patches in S1 Fig) and five shelters on the right end of roads (dark blue patches in S1 Fig). Gridded roads (white patches) follow one of road structures in many real-world cities [1–5]. Traffic signals exist at every entrance to intersections. Shelters are safe places where residents are protected during the disaster. Each shelter has its capacity, a maximum room to accommodate evacuees. When a shelter gets to the full capacity, a shelter closes and blocks evacuees from entering.


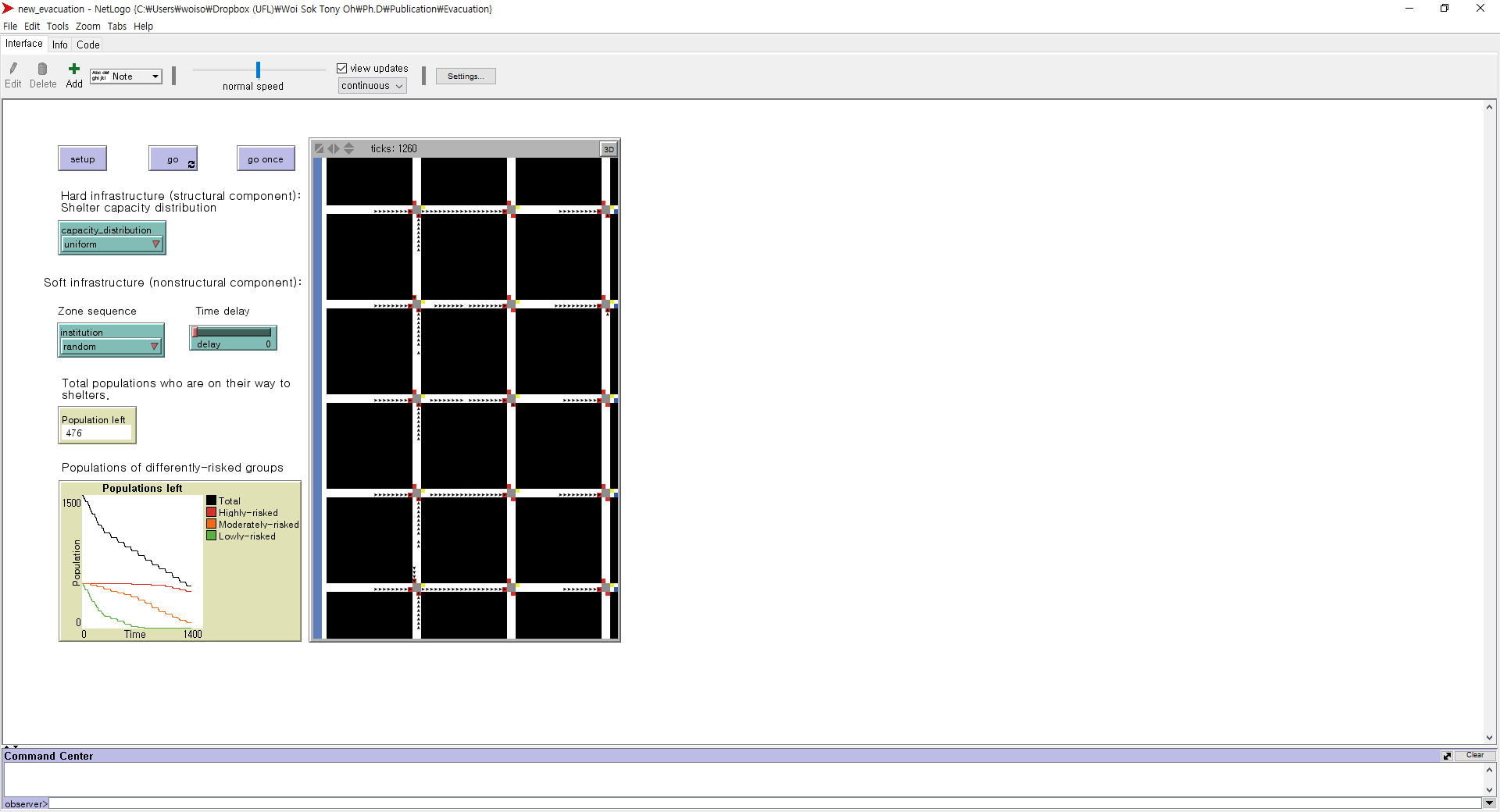


**S1 Fig.** An overview of the NetLogo interface in our ABM. Evacuating cars (black arrows) are agents in our model, initially located in the sources (see Fig 1 in the main text). White patches are roads, grey patches are intersections, and green/yellow/red patches are traffic signals. Light blue patches (left column) indicate the waterfront vulnerable to floods, and dark blue patches on the right end are safe shelters. You can select different hard infrastructural and institutional settings in the choosers and a slider at the left.

Agents are configured to be evacuees on their vehicles, leaving their residence without staying at home or going upper floors during/before the flooding events. An evacuee gets more risked by the flooding events if he/she lives close to the left side (close to the waterfront). Otherwise, he/she becomes less risky as it is easier for him/her to go to a safe shelter.

**S1 Table.** Descriptions, units, and values of model parameters.

| **Par** | **Descriptions** | **Values** |
| --- | --- | --- |
| *p* | Initial population | 1500 |
| *t_g_* | Time interval for the green traffic lights (ticks) | 17 |
| *t_y_* | Time interval for the yellow traffic lights (ticks) | 3 |
| *t_r_* | Time interval for the red traffic lights (ticks) | 60 |
| *f* | Percentage of residents who knows which shelter is the closest one (%) | 90 |
| *σ_1_* | Standard deviation for the strongly non-uniform shelter capacity before the normalization | 1 |
| *σ_2_* | Standard deviation for the moderately non-uniform shelter capacity before the normalization | 2 |

**S2 Table.** Descriptions of state variables.

| **State variables** | **Descriptions** |
| --- | --- |
| *d* | Averaged evacuation duration of all agents |
| DOU | Gini coefficient for degrees of suffering (**s**) of all agents (Degree of unfairness) |

We focus on how capacities (maximum number of people a shelter can take) are configured in five shelters as a hard infrastructure setting. Given that we have 1500 evacuees, the total capacity is 1515 (an additional 1% capacity for numerical errors). When shelter distribution is *uniform* (U), five shelters have an equal capacity of 303. When shelter distribution is *strongly non-uniform* (SN), the shelter capacities are highly concentrated in the center. It sharply decreases as a shelter is far from the center (max shelter capacities are 41, 304, 825, 304, and 41 from shelters on the top to those on the bottom)—like a normal distribution with a low standard deviation. When shelter distribution is *moderately non-uniform* (MN), shelter capacities evenly distributed over five shelters (max shelter capacities are 204, 337, 433, 337, and 204 from shelters on the top to the bottom)—like a normal distribution with a high standard deviation. Figs 1B, C, and D in the main text display the shelter capacity distributions.

We test simultaneous and staged evacuation as institutional arrangements. *Simultaneous evacuation* is when agents in all zones begin evacuation at the same time (Fig 2A in the main text). *Staged evacuation* divides the area into several zones and prioritizes certain areas to evacuate first. Particularly, staged evacuation has two internal features: zone sequence and time delay. Zone sequence refers to when people in each zone are allowed to start the evacuation. Time delay is the time difference between each zone in the staged evacuation. Our model has H (high-risk), M (moderate-risk), and L (low-risk) zones, and thereby six possible zone sequences exist (HML, HLM, MHL, MLH, LHM, and LMH). For example, the more an agent is risked (live closer to the waterfront), the more he/she is prioritized in the HML strategy (Fig 2B in the main text).

Our ABM has conceptual units, for example, time units in tick and distance units in patch.

1. **Process overview and scheduling**

We select a shelter capacity distribution and simultaneous/staged evacuation planning at the initial setup stage.

In each time step, agents and the environment go through the following processes:

1. Traffic signs are updated.

We have four traffic signals at each intersection (see S2 Fig). A signal on the west side is initially green and turned on for 17 ticks. The signal becomes yellow afterward for 3 ticks and then turns red for 60 ticks. Once this signal turns red, a signal on the north side becomes green. Signals repeatedly become green in a clockwise direction.


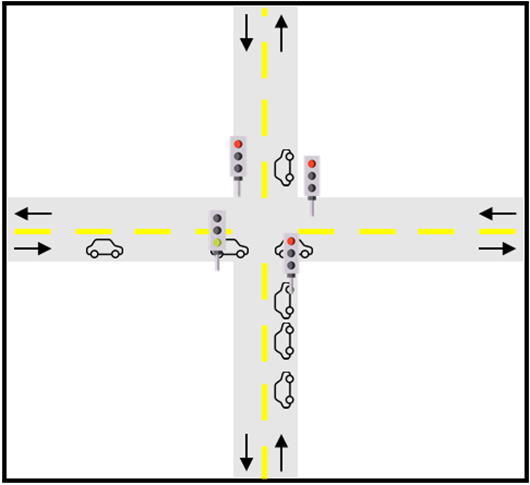


**S2 Fig.** A zoomed view of intersections. Roads are two-way in opposite directions, and signals exist in front of the intersection.

1. Agents evacuate.

- Each agent leaves from the source one at a time to its destination. The time when an agent is allowed to leave differs depending on the initial setting of zone sequence and time delay.
- An agent evacuates toward the assigned shelter after leaving its source. If the agent has several shortest routes to the targeted shelter, he/she chooses one of the shortest routes at random; the agent randomly decides whether to go straight or turn to other directions at the intersections.
- Evacuating cars move based on the simplified Nagel-Schreckenberg traffic model with a constant speed of 1 patch per tick [6]. An agent stops (i) when a car is stopped in front, (ii) when a traffic signal is yellow/red, or (iii) when the agent arrives at the vacant shelter.

1. Shelters get filled with people.

- When an agent arrives at a shelter, he/she finishes the evacuation and disappears from the model environment.
- An agent cannot enter a shelter at the maximum capacity. If shelter capacity reaches the maximum, a road connected to the shelter is blocked (black patch).
- Since evacuees cannot check social media or the internet during the evacuation, agents do not know whether a shelter is still opened or closed until they arrive at shelters. If an agent arrives at the targeted shelter with a full capacity, he/she gets information about the closest vacant shelter. He/she continues the evacuation process to a new target (full information).

When all evacuees move into shelters, a simulation ends. Then, the model reports the average evacuation duration and DOU of all agents.

1. **Design concepts**

*Basic principles*. This ABM is conceptual and drops secondary components in the evacuation. This dimensional reduction clarifies complex interactions and their underlying mechanisms by focusing on the primary components we are interested in.

*Emergence*. Our model produces evacuation duration and DOU. We average evacuation durations of 500 simulations to calculate evacuation efficiency. We define fairness to be a condition *when agents equally suffer from floods*. We first calculate a degree of suffering for agent *i* (*s_i_*), which is a multiplication of evacuation duration (*t_i_*) and flood risk at agent *i*’s residence (*r_i_*; a distance between residence and shelters in west-east direction)—*s_i_ = t_i_ ∙ r_i_*. Then, DOU is estimated by finding Gini coefficients of *s_i_* for 1500 agents and averaging Gini coefficients from 500 simulations (Equation 2 in the main text).

*Adaptation*. Here, one adaptive trait is a changing destination when the targeted shelter is already full. If an agent arrives at the targeted shelter and finds it to be full, he/she changes the destination to the closest shelter with available rooms.

*Objectives*. Each agent aims to evacuate to a safe shelter as soon as possible. However, an agent does not compare the time with others. An agent’s route selection is random rather than utility-based once he/she chooses a shelter at the initial setup.

*Sensing*. Initially, *f* percent of agents have full information about the closest shelter. The rest randomly chooses one of five shelters as their destinations. We assume that all the shelters share information about room availability. Suppose an agent goes to a shelter at full capacity (an agent does not have any information about a shelter’s vacancy until he/she arrives). In that case, he/she is informed where the closest vacant shelter is and moves to the place (full information).

*Interaction*. Evacuating agents interact based on the Nagel-Schreckenberg traffic model [6]. When other agents fill a shelter where the agent targets to evacuate before his/her arrival, he/she has to change the destination (indirect interaction).

*Stochasticity*. 10 percent of agents randomly choose their shelters regardless of the distance. Once agents start their evacuation, they randomly choose which route to take.

1. **Initialization**

Initially, 1500 residents are equally distributed over all sources. Agents are equally located in the sources at *t =* 0 (yellow circles in Fig 1A of the main text) like source-sink approaches in the network-based models [7]. Each source initially has 50 agents—1500 agents in total. In sources of the vertical roads, half of the agents start their evacuation in the north direction, and the rest start in the south direction. *f* percent of evacuees have full information about which shelter is the closest to their residence, while the rest have imperfect information and randomly choose their shelters to evacuate. We also choose shelter capacity, zone sequence (simultaneous and staged evacuation), and time delay (if staged evacuation) at the setup stage.

**References (only for Supporting Information)**

1. Chen X, Zhan FB. Agent-based modelling and simulation of urban evacuation: relative effectiveness of simultaneous and staged evacuation strategies. J Oper Res Soc. 2008;59: 25–33. doi:10.1057/palgrave.jors.2602321

2. Watanabe D. A Study on Analyzing the Grid Road Network Patterns using Relative Neighborhood Graph. The Ninth International Symposium on Operations Research and Its Applications (ISORA’10). Chengdu-Jiuzhaigou, China; 2010. Available: http://citeseerx.ist.psu.edu/viewdoc/download?doi=10.1.1.645.6992&rep=rep1&type=pdf

3. Miyagawa M. Optimal hierarchical system of a grid road network. Ann Oper Res. 2009;172: 349–361. doi:10.1007/s10479-009-0630-4

4. Hu X, Tao C, Hu Y, Tao V. Automatic road extraction from dense urban area by integrated processing of high resolution imagery and LIDAR data. International Society for Photogrammetry and Remote Sensing. 2004. pp. 320–324. Available: https://www.researchgate.net/publication/228803852

5. Wang F. Urban Population Distribution with Various Road Networks: A Simulation Approach. Environ Plan B Plan Des. 1998;25: 265–278. doi:10.1068/b250265

6. Nagel K, Schreckenberg M. A cellular automaton model for freeway traffic To cite this version : cellular. J Phys. 1992;2: 2221–2229.

7. Zhao X, Feng Z, Li Y, Bernard A. Evacuation Network Optimization Model with Lane-Based Reversal and Routing. 2016;2016.
